# The global leadership initiative on malnutrition criteria for the diagnosis of malnutrition in patients with chronic liver diseases: a systematic review and meta-analysis

**DOI:** 10.3389/fnut.2025.1612417

**Published:** 2025-06-19

**Authors:** Zhiming Wang, Yuping Cao, Yumei He, Menghao Hao, Shiyan Wu, Lu Li, Qiong Wang, Xiaobin Sun, Liping Wu

**Affiliations:** ^1^Department of Gastroenterology, The Affiliated Hospital of Southwest Jiao Tong University, The Third People’s Hospital of Chengdu, Chengdu, China; ^2^School of Clinical Medicine, Southwest Medical University, The Affiliated Hospital of Southwest Medical University, Luzhou, China; ^3^North Sichuan Medical College, Nanchong, China; ^4^School of Medicine, Southwest Jiao Tong University, The Affiliated Hospital of Southwest Jiao Tong University, The Third People's Hospital of Chengdu, Chengdu, China

**Keywords:** GLIM, SGA, chronic liver disease, meta-analysis, systematic review

## Abstract

**Background:**

Malnutrition in patients with chronic liver disease (CLD) is linked to increased mortality and a high risk of morbidity. Assessing the nutritional status of patients with CLD is challenging. The Global Leadership Initiative for Malnutrition (GLIM) offers a novel diagnostic framework for malnutrition. However, the efficacy of GLIM in CLD patients has not been validated.

**Methods:**

A systematic review and meta-analysis were conducted to evaluate the utility and diagnostic accuracy of the GLIM criteria in adult patients with CLD, involving a search of seven databases for relevant studies. The evaluation of quality was conducted with the QUADAS-2 tool.

**Results:**

The analysis included a total of five studies. Sample size ranged from 109 to 406 among different studies. According to the GLIM criteria, around 21.2 to 69.9% of individuals were identified as having malnutrition. Simultaneously, the subjective global assessment (SGA) detected malnourished patients ranging from 35.0 to 86.0%. Five studies compared the GLIM with the SGA. The nutritional assessment process in the studies was not clear according to the QUADAS-2 tool. The overall specificity of the meta-analysis was 85.8% (95% CI: 82.5–88.7%) and the overall sensitivity was 49.1% (95% CI: 45.5–52.8%).

**Conclusion:**

This is the first systematic review and meta-analysis on GLIM criteria, SGA and CLD patients. The applicability and reliability of the GLIM criteria in CLD patients remain constrained. Furthermore, certain validation studies that are parallel and predictive may have methodological limitations. Additional research is needed to confirm the applicability of the GLIM criteria in patients with CLD.

**Systematic review registration:**

PROSPERO, CRD420251010347.

## 1 Introduction

Malnutrition is usually associated with social, economic, and demographic factors, but most importantly, a patient’s disease can negatively affect nutritional status. Thus, disease-related or in-hospital malnutrition is highly prevalent, while malnutrition also affects the disease (1, 2). The clinical significance of malnutrition in individuals with liver conditions was first acknowledged in 1964 by Turcotte and Child, who categorized disease severity using five criteria: serum bilirubin level, serum albumin level, presence of ascites, encephalopathy, and nutritional status (3). Reduced food consumption is a significant factor contributing to malnutrition and has been shown to have a negative impact on prognosis, especially in terms of protein intake (4, 5). Sarcopenia, the loss of muscle mass and function, has been identified as a significant indicator of negative outcomes in patients with liver disease according to recent findings (6, 7).

It is estimated that 1.8 million deaths were attributed to chronic liver disease (CLD) and hepatocellular carcinoma in 2015 (8). Malnutrition in patients with advanced CLD has been linked to unfavorable clinical outcomes, including bacterial infections and postoperative complications, as well as a diminished quality of life and reduced survival time (9). Physical and functional alterations in patients with CLD directly affect nutritional status, rendering malnutrition a prevalent condition, impacting 20 to 50% of patients (10).

Malnutrition and nutritional deficiencies should be recognized when treating patients with CLD. Commonly used nutritional evaluation tools include the Subjective General Assessment (SGA), the Royal Free Hospital-global assessment (RFH-GA), and nutritional screening tools include the Nutritional Risk Screening Tool 2002 (NRS-2002) and the Royal Free Hospital Nutritional Prioritization Tool (RFH-NPT) (10–12).

The SGA is a malnutrition assessment tool commonly used worldwide since its inception (13), and is widely used in the nutritional assessment of patients because of its ease of use and high reproducibility. In the absence of a gold standard, some studies have considered it as the most validated tool for assessing malnutrition in the hospital setting (14–16). The SGA consists of eight indices categorized into grades A, B, and C, with patients with at least five indices in grades B or C being categorized, respectively, as moderate or severe malnutrition, respectively (11, 12, 17). However, agreement between SGA and other methods of assessing nutritional status, such as BMI and mid-arm muscle circumference, is low (10, 18). Although these above tools are often used for clinical nutritional risk assessment, complications in patients with CLD, such as sodium retention, may affect the scoring of these tools (19). The use of these tools in patients with chronic malnutrition requires further validation.

The Global Leadership Initiative on Malnutrition (GLIM) criteria, published in 2019, is a new framework that has been created to establish consistent diagnostic criteria for malnutrition and enhance patient outcomes in clinical settings (20–22). A meta-analysis indicates that the GLIM criteria have high diagnostic accuracy for distinguishing malnutrition and may have the potential to be used as a gold standard for diagnosing malnutrition in clinical practice (23). GLIM divides the assessment of malnutrition in hospitalized patients into two steps, namely nutritional risk screening and diagnostic assessment. Malnutrition is determined by the GLIM through the presence of at least one phenotypic criterion such as low body mass index (BMI), involuntary weight loss, or decreased muscle mass, along with etiologic criterion related to disease burden/inflammatory condition and reduced food intake (20–22). The GLIM criteria, utilized as a standardized nutritional assessment tool, is anticipated to decrease global variability in diagnosing malnutrition. Furthermore, the GLIM provide a thorough assessment of malnutrition, allowing healthcare providers to pinpoint the factors that lead to sarcopenia and impact the clinical outcomes of CLD patients (20–22).

In clinical practice, GLIM criteria have shown promising results in validation studies. However, methodological issues have been identified in published studies. A recent assessment of the validity of the GLIM criteria found that only 25% detailed the process of determining sample size and only 3% provided data that were considered reliable. Therefore, there is a need for more high-quality validation studies (24). Moreover, individual studies may possess insufficient power to ascertain overall effects. Therefore, this current study and meta-analysis were conducted to evaluate the use and accuracy of the GLIM criteria in individuals with chronic liver disease.

## 2 Methods

### 2.1 Study design

The meta-analysis followed the Preferred Reporting Items for Systematic Reviews and Meta-analysis of Diagnostic Test Accuracy Studies (PRISMA-DTA) guidelines for conducting systematic reviews and meta-analyses of diagnostic test accuracy studies (25). The research plan was recorded on PROSPERO (CRD420251010347), with search strategies and methodologies determined prior to commencing the study.

### 2.2 Search strategy

A bibliographic search was performed across seven databases (Web of Science, PubMed, Embase, Science Direct, Scopus, SciELO, and China National Knowledge Infrastructure). The studies included were published within the period from January 01, 2019, to March 12, 2025. The search algorithm utilized encompassed the following terms: (“Global Leadership Initiative on Malnutrition” OR “GLIM diagnosis” OR “GLIM criteria” OR “GLIM framework” OR “GLIM”) AND (“chronic liver diseases” OR “CLD” OR “hepatic disease” OR “cirrhosis”) (Supplementary Table S1). After importing all references and summaries into a reference management tool, duplicates were removed, and an Excel file was created for the study selection process.

### 2.3 Study selection

The inclusion criteria were longitudinal observational or cross-sectional studies that were written in English or Chinese of adult (18 years of age or older) patients with CLD of any etiology with a diagnosis of malnutrition within 72 h of admission using the GLIM criteria. The exclusion criteria were non-hepatic active malignancies, liver transplantation during the follow-up period, a history of organ transplantation, and life-threatening comorbidities, such as heart, respiratory, and renal failure. Studies lacking nutritional assessment data or complete texts were excluded.

Two researchers independently extracted and screened all titles and abstracts obtained from the literature search. They received instruction on how to choose references and demonstrated a strong level of agreement in deciding which articles to include or exclude. References were chosen based on their titles and abstracts, with full-text articles then acquired and study selection criteria re-verified. Two researchers identified potentially relevant studies and performed data extraction, and if there was disagreement between researchers, this was discussed and agreed with a third researcher.

### 2.4 Nutritional diagnosis

The GLIM criteria include two steps: risk screening and diagnosis. First, nutritional screening tool, such as NRS-2002 was used to screen patients at risk of malnutrition (20). Next, a malnutrition diagnosis was performed on patients at risk of malnutrition. Patients who meet at least one of the phenotypic criteria (weight loss, low body mass index, and reduced muscle mass) and at least one of the etiologic criteria (reduced food intake and disease burden/inflammation) can be diagnosed with malnutrition in this study (20). Serum albumin can be used as supportive proxy measures of inflammation/disease burden, as reported by the GLIM consensus (20). In this study, patients with serum albumin <35 g/L were defined as having hypoproteinemia and were considered to have a disease burden (20). SGA consisted of eight parameters, weight and dietary intake changes, gastrointestinal symptoms, functional capacity, nutritional burden-related disease, loss of subcutaneous fat, muscle wasting and the presence of oedema/ascites, where weight was still calculated as dry weight (13). SGA was performed by trained nutritionists using the criteria described in previous studies, where malnutrition was defined as SGA categories B or C. Diagnostic criteria for malnutrition are shown in Supplementary Figure S1.

### 2.5 Data extraction

The variables of interest included study characteristics (author, year of publication, country, duration of follow-up, age, sample size, gender, Apache score, and tools for nutritional assessment), adherence to GLIM criteria (combination of phenotypic and etiological criteria), nutritional evaluation outcomes, and metrics for diagnostic test accuracy, such as true positives, false positives, true negatives, and false negatives. The reviewers utilized a standardized data collection instrument to extract the data. The source of information was the full text of the document. If the original article lacked detailed data, attempts were made to contact the researcher via email on two occasions to obtain the missing information.

### 2.6 Assessment of article quality

All original articles were assessed using the quality evaluation instrument created by the National Institutes of Health for observational cross-sectional and cohort studies. The methodological quality of each original article was assessed, employing the Quality Assessment Tools for Diagnostic Accuracy Studies (QUADAS-2) tool (26).

### 2.7 Synthesis and statistical analysis

The tables and main text contain the pertinent findings from each study. Data pertaining to test performance, including true positives, false positives, true negatives, and false negatives, were extracted from each individual study. Estimates of test performance were derived from the available sensitivity and specificity data reported within the manuscript.

The meta-analysis utilized the bivariate random-effects model. Visual exploration of the diagnostic accuracy for each test was accomplished through forest plots of sensitivity and specificity. Performance of the GLIM criteria was deemed satisfactory if the confidence interval’s lower limit for sensitivity and specificity exceeded 80% (27, 28). Additionally, we generated a summary receiver operating characteristic curve (SROC). The analyses utilized RevMan 5.2 (The Cochrane Co-operation, Oxford, UK), Stata 12 (Stata Corporation, College Station, TX, United States), and Meta-DiSc 2.0 (XI Cochrane Colloquium, Barcelona, Spain).

## 3 Results

**Figure 1:**
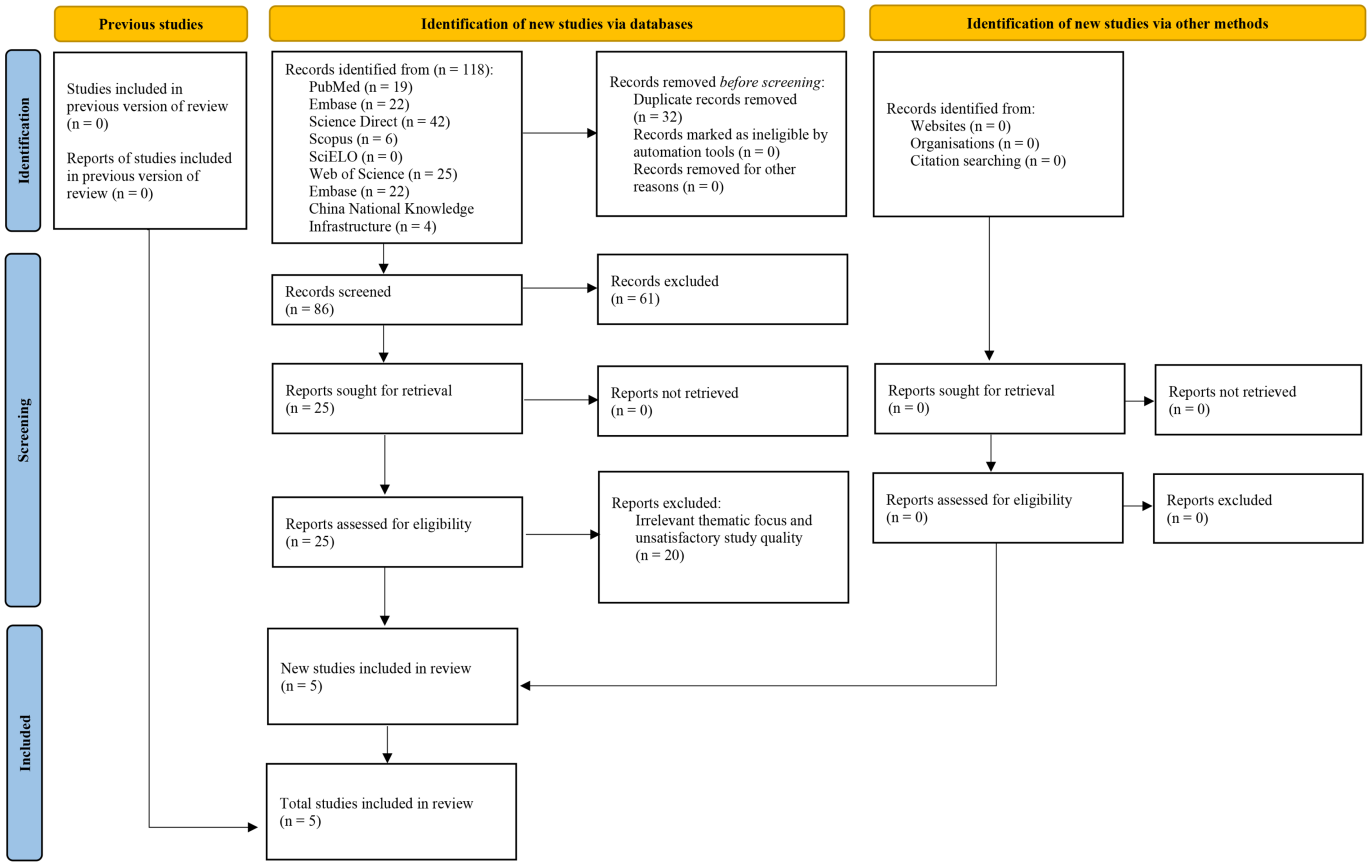
Flow diagram of the search strategy and study selection process.

This review included five articles (29–33) out of a total of 118 references found (Figure 1). Sample size ranged from 109 to 406 among different studies (Table 1).

**Table 1:** Characteristics of the included studies.

Study	Design	Criteria used and nutritional assessment tool	Sample size Male/female age
Miwa 2022, Japan (29)	Retrospective observational study	SGA, RFH-GA, and GLIM criteria	*n* = 406 274/228 74 (IQR: 66–79)
Wu 2022, China (30)	Prospective observational study	NRS-2002, RFH-NPT, SGA, and GLIM criteria	*n* = 113 83/30 56.81 ± 10.90
Santos 2022, Brazil (31)	Retrospective observational study	SGA and GLIM criteria	*n* = 152 101/51 52.0 (IQR: 46.5–59.5)
Wu 2022, China (32)	Prospective cohort study	SGA and GLIM criteria	*n* = 109 78/31 56.36 ± 10.39
Jiang 2024, China (33)	Retrospective observational study	NRS-2002, SGA, and GLIM criteria	*n* = 335 215/120 55.70 ± 13.40

SGA, subjective global assessment; RFH-GA, Royal Free Hospital-global assessment; GLIM, Global Leadership Initiative on Malnutrition; IQR, interquartile range; NRS-2002, Nutritional Risk Screening 2002; RFH-NPT, Royal Free Hospital-Nutritional Prioritizing Tool.

### 3.1 Quality assessment

After using the National Institutes of Health tool, it was found that four studies (29–32) showed high quality, as detailed in Table 2. A comparison of the GLIM criteria and the SGA was conducted across five studies (29–33). Moreover, after using the QUADAS-2 tool, it was found that the nutritional evaluation was lacking in several studies, as shown in Table 3.

**Table 2:** Use of the National Institutes of Health tool for the quality assessment of observational studies.

Major components	Miwa 2022, Japan (29)	Wu 2022, China (30)	Santos 2022, Brazil (31)	Wu 2022, China (32)	Jiang 2024, China (33)
Question statement	Yes	Yes	Yes	Yes	Yes
Population statement	Yes	Yes	Yes	Yes	Yes
Participation	Yes	Yes	Yes	Yes	Yes
Eligibility criteria	Yes	Yes	Yes	Yes	Yes
Sample size justification	Yes	Unclear	Yes	Unclear	Unclear
Exposure before outcome	No	No	No	No	No
Follow-up	Yes	Yes	Yes	Yes	Yes
Levels of exposure	Not applicable	Not applicable	Not applicable	Not applicable	Not applicable
Definity exposure	Not applicable	Not applicable	Not applicable	Not applicable	Not applicable
Multiple exposure assessment	Not applicable	Not applicable	Not applicable	Not applicable	Not applicable
Definity outcome	Yes	Yes	Yes	Yes	Yes
Blinded outcome	Not reported	Unclear	Not reported	Unclear	Unclear
loss of follow-up	Yes	Not reported	Yes	Not reported	No
Confounding variables measured	Yes	Yes	Yes	Unclear	Unclear
Quality rating	Good	Good	Good	Good	Fair

**Table 3:** Quality assessment and risk of bias using the QUADAS-2 tool.

References	Miwa 2022, Japan (29)	Wu 2022, China (30)	Santos 2022, Brazil (31)	Wu 2022, China (32)	Jiang 2024, China (33)
Sample	Yes	Yes	Yes	Yes	Yes
Case–control avoid	Yes	Yes	Yes	Yes	Yes
Eligibility	Yes	Yes	Yes	Yes	Yes
Patient selection	Low	Low	Low	Low	Low
Patients and review	Low	Low	Low	Low	Low
Blinded index test results	Unclear	Unclear	Unclear	Unclear	Unclear
Pre-specified threshold	Yes	Yes	Yes	Yes	Yes
*Index test bias*	Unclear	Unclear	Unclear	Unclear	Unclear
Index test and review	Low	Low	Low	Low	Low
Reference standard likely to correctly classify the condition	Yes	Yes	Yes	Yes	Yes
Blinded reference	Unclear	Unclear	Unclear	Unclear	Unclear
Standard results	Low	Low	Low	Low	Low
*Reference standard bias*	Low	Low	Low	Low	Low
Condition, reference standard, and review	Not applicable	Not applicable	Not applicable	Not applicable	Not applicable
Interval between index test and reference	Unclear	Unclear	Unclear	Unclear	Unclear
All patients received a reference standard	Yes	Yes	Yes	Unclear	Unclear
All patients received the same reference standard	Yes	Yes	Yes	Yes	Yes
All patients included in the analysis	Yes	Yes	Yes	Yes	Yes
*Patient flow bias*	Low	Low	Low	Low	Low

### 3.2 GLIM criteria assessment

Evaluation of the GLIM phenotypic criteria involved assessing weight loss, muscle mass, and low BMI, while the etiologic criteria focused on disease burden and inflammation based on clinical condition (Table 4). Only one of the studies evaluated all five GLIM criteria (34).

**Table 4:** GLIM criteria used in the studies.

Study	Phenotypic			Etiologic		Diagnosis using the GLIM criteria
	Weight loss	Low BMI	Reduced muscle mass	Reduced food intake or assimilation	Inflammation	
Miwa 2022, Japan (29)	Yes	Yes	Yes	Yes	Serum CRP levels	1 Phenotypic + 1 Etiologic
Wu 2022, China (30)	Yes	Yes	Yes	Yes	Determined based on chronic inflammatory state	1 Phenotypic + 1 Etiologic
Santos-12022, Brazil (31)	No	No	AMA	MELD-Na	Level of serum albumin	AMA + MELD-Na
Santos-22022, Brazil (31)	No	No	AMA	Child-Pugh	Level of serum albumin	AMA + Child-Pugh
Wu 2022, China (32)	Yes	Yes	Yes	Yes	Determined based on chronic inflammatory state	1 Phenotypic + 1 Etiologic
Jiang 2024, China (33)	Yes	Yes	Yes	Yes	Serum albumin <35 g/L	1 Phenotypic + 1 Etiologic

GLIM, Global Leadership Initiative on Malnutrition; BMI, body mass index; CRP, C-reactive protein; AMA, arm muscle area.

### 3.3 Prevalence of malnutrition

The prevalence of malnutrition, as assessed with the SGA in five studies, ranged from 35.0 to 86.0%. In contrast, assessments utilizing the GLIM criteria indicated a prevalence ranging from 21.2 to 69.9% (Table 5).

**Table 5:** Nutritional status.

Study	GLIM criteria		SGA	
	Well-nourished	Malnourished	Well-nourished	Malnourished
Miwa 2022, Japan (29)	78.8%	21.2%	65.0%	35.0%
Wu 2022, China (30)	34.9%	65.1%	45.0%	55.0%
Santos-12022, Brazil (31)	69.7%	30.3%	36.8%	63.2%
Santos-22022, Brazil (31)	71.1%	28.9%	36.8%	63.2%
Wu 2022, China (32)	30.1%	69.9%	42.5%	57.5%
Jiang 2024, China (33)	65.7%	34.3%	14.0%	86.0%

GLIM, Global Leadership Initiative on Malnutrition; SGA, Subjective Global Assessment.

### 3.4 Concurrent validation

**Figure 2:**
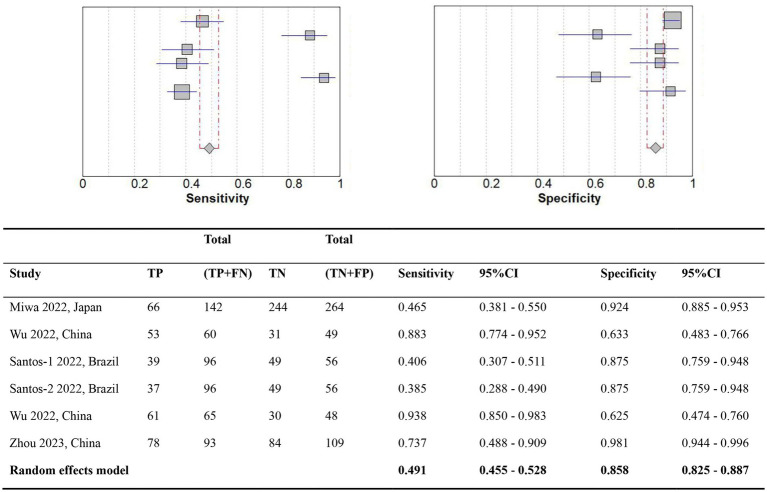
Forest plot of the diagnostic accuracy comparing GLIM vs. SGA. TP, True positives; TN, True negatives; FN, False negatives; FP, False positives; CI, Confidence interval.

**Figure 3:**
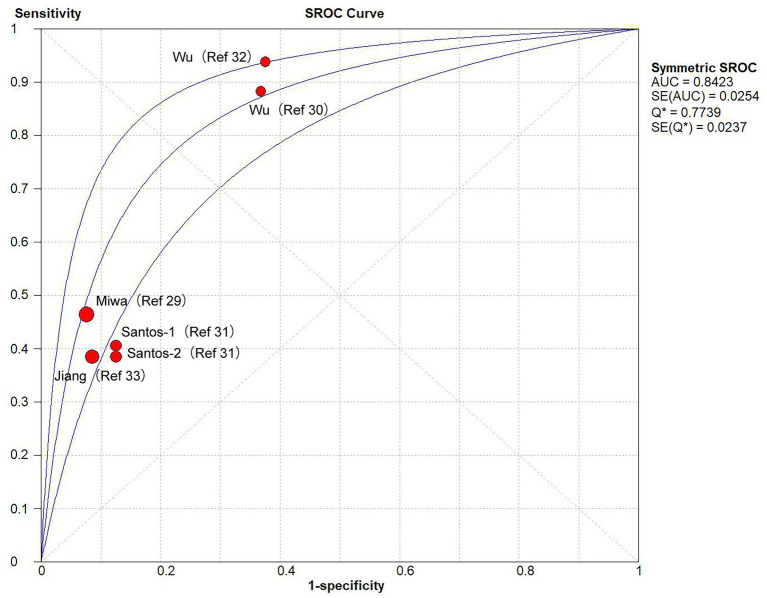
Receiver operating characteristic curve of the diagnostic accuracy of studies comparing GLIM vs. SGA.

A comparison of the GLIM criteria with the SGA, across five studies, involved a total sample size of 1,115 participants. Figures 2, 3 illustrate the meta-analysis results, which reported an overall sensitivity of 49.1% (95% CI: 45.5–52.8%) and an overall specificity of 85.8% (95% CI: 82.5–88.7%).

### 3.5 Predictive validity

The main outcomes measured were mortality, and length of hospital stay. Research conducted by Miwa et al. (29) found an increased risk of mortality for individuals identified with malnutrition using the GLIM criteria (HR 3.16, 95% CI 2.26–4.37, *p* < 0.001), whereas the SGA and RFH-GA did not predict mortality of patients without hepatocellular carcinoma (HCC). Wu et al. (30) compared malnourished and well-nourished patients based on length of hospital stay. The results showed that patients diagnosed as malnourished using both SGA and GLIM criteria had a longer hospital stay than well-nourished patients. A study by Jiang et al. (33) showed that diagnosis based on the GLIM criteria had a better performance in predicting in-hospital mortality in malnourished and well-nourished patients (AUC = 0.666, 95% CI 0.549–0.782, *p* = 0.008) than SGA (AUC = 0.505, 95% CI 0.384–0.627, *p* = 0.932).

## 4 Discussion

The GLIM framework, proven practical for diagnosing malnutrition, is also applicable to patients with CLD (20–22). At present, no definitive standard exists for nutritional assessment in CLD patients, leading to variability in nutritional assessment based on populations, medical diagnosis, institution protocols, and the expertise of healthcare providers (35). A meta-analysis performed by Díaz et al. investigated the use and validity of the GLIM criteria in patients admitted to the ICU, however, there are no meta-analyses on CLD patients and the GLIM (36). Our results synthesize previous studies and show that the GLIM is highly specificity and predictive in identifying malnourished patients with CLD, and is therefore may be a suitable tool for diagnosing such patients for nutritional status.

Malnutrition is now recognized as a non-negligible complication in patients with CLD, and timely, precise nutritional assessment of CLD patients is crucial for directing clinical nutritional interventions, thereby enhancing patient prognosis, which holds substantial importance in clinical practice. However, nutritional assessment of patients with CLD is challenging due to fluid retention and impaired hepatic synthetic function (34, 37). Fluid retention is very common in patients with CLD, and the assessment of fluid retention is an important component of the SGA (20), which may explain the high rate of malnutrition described by the SGA in this study. The SGA includes many subjective assessments for the nutritional diagnoses. However, subjective assessment of reduced muscle mass in patients with CLD may be challenging. Fat-free mass (FFM) by Bioelectrical impedance analysis (BIA) and the total lean mass by dual energy x-ray absorptiometry (DEXA) may be influenced by ascites or edema (38, 39). Cross-sectional skeletal muscle area (SMA) at the L3 level can also be affected by overhydration in these patients (40). Future studies should validate specific cutoff points (41) and illustrate the impact of fluid retention in patients with CLD (40). On the other hand, GLIM combinations, including BMI and low muscle mass, assessed by reliable methods as a phenotypic criterion could be useful (31).

The first step in diagnosis using the GLIM framework is to perform nutritional risk screening, and a study by Bannert et al. (42) found differences in the sensitivity of different risk screening tools such as the NRS-2002, the RFH-NPT, and the MUST. Of note, the RFH-NPT had remarkably high sensitivity in patients with cirrhosis (43). The RFH-NPT is a tool that was developed specifically for patients with chronic liver disease and should perhaps be prioritized in future studies of nutritional risk screening in patients with CLD (44). To date, researchers have not clearly identified the best nutritional screening tool for use in patients with CLD. Therefore, in future studies, the use of GLIM for diagnosis should be guided by evidence-based medicine to select the appropriate screening tool.

The effectiveness of the GLIM is influenced by the way in which the phenotypic and etiologic criteria are combined (45). Among the five studies included in our current meta-analysis, only one study used all five GLIM criteria and tested 36 different combinations of GLIM (31). Prior research has indicated that incorporating muscle mass assessment into the GLIM framework improves its effectiveness (46). Therefore, different combinations of the GLIM should always include an assessment of muscle mass (35).

Our meta-analysis during the simultaneous validation of the GLIM against the SGA revealed a lack of accuracy in the included studies, with sensitivity falling below the 80% threshold of the confidence interval’s lower bound (1). This outcome may be attributed to the heterogeneity observed among study results and methodological constraints, including inadequate sample sizes, reliance on anthropometric measurements for assessing muscle mass, and potential selection bias (24). The findings of our study, which revealed high specificity and low sensitivity of the GLIM criteria, align with the results reported by Allard et al. (47). The GLIM criteria propose the presence of chronic and acute disease-related inflammation, such as chronic heart failure, as an etiologic criterion for the diagnosis of malnutrition. Considering the prevalence of chronic disease-related inflammation in patients hospitalized with CLD and the fact that bedside clinicians may use this criterion as an etiologic criterion in the absence of CRP measurements, it would make sense to further assess the validity of the GLLM while including the presence of these acute and chronic diseases as variables. In our meta-analysis, since all patients had an acute or chronic active disease burden (48), to be more specific, we chose to use CRP, Serum albumin, as one of the ancillary laboratory measures of inflammation, as recommended by the GLLM guidelines. This may be one of the reasons for the higher specificity and lower sensitivity. Sensitivity is inversely proportional to the false negative rate. A low sensitivity GLIM may lead to more false-negative results, meaning that patients who are actually positive may be misclassified as negative, potentially obscuring the true disease state. Therefore, the application of GLIM for assessing nutritional status in clinical practice necessitates careful consideration of etiologic criteria. Moreover, we emphasize the importance of adhering to scientific methodology, such as prospective study design, reliable sample size, and appropriate statistical analysis, to ensure the comprehensive validation of the GLIM criteria.

Regrettably, conducting a predictive validity meta-analysis on malnutrition using the GLIM criteria and clinical outcomes was not feasible due to the scarcity of studies reporting outcomes like mortality, potentially introducing spectrum bias. Therefore, future studies should include as many prospective studies as possible to ensure that the GLIM criteria are properly validated in patients with CLD (49).

The methodology used in this study has significantly reduced the potential for bias. Selection bias was avoided by the utilization of multiple databases, stringent selection criteria, and data analysis by a team of three researchers. The execution of searches in two languages, Chinese and English, diminished language bias. Publication bias could not be discerned due to an insufficient number of published studies, precluding the performance of statistical analyses such as Egger’s test. The included studies were of high quality, resulting in a low risk of bias. However, as previously noted, there are methodological aspects that require refinement for future validity research (24).

One advantage of this study is that it conducted a meta-analysis for diagnosing malnutrition, comparing GLIM with SGA. This allows for an assessment of the GLIM criteria’s validity in patients with CLD. However, a primary limitation was the restricted number of studies included, which limited the potential for conducting meaningful subgroup analyses. Our search strategy, confined to indexed journal databases, should be expanded in the future to include gray literature sources, provided they meet scientific methodological standards.

This study is believed to be the first meta-analysis evaluating the accuracy of the GLIM criteria in patients with CLD. Our research showed that only a few studies have used the GLIM criteria in patients with CLD, and most of them did not adequately evaluate the tool’s validity due to poor methodological quality and potential bias. The GLIM criteria show promise for nutritional diagnosis in CLD patients, as long as the methods are clear and followed, based on the collected data and proven predictive accuracy. Hence, further research is needed to confirm the validity of the GLIM criteria in patients with CLD, with an emphasis on improving methodological quality. Validation research on the GLIM standards in patients with chronic liver disease should focus on increasing diversity among participants by utilizing a multicenter approach and a larger sample size. Furthermore, subsequent studies should compare other validated nutritional assessment tools and delineate the application of the five GLIM criteria in combination.

## 5 Conclusion

The use of GLIM in patients with CLD has not been widespread, and multiple validation studies that assess both concurrent and predictive validity show limitations in methodology. Nonetheless, our meta-analysis and demonstrated predictive validity suggest that this tool could be beneficial. Nevertheless, these results require confirmation through studies that utilize a strict scientific approach.

## Data Availability

The original contributions presented in the study are included in the article/Supplementary material, further inquiries can be directed to the corresponding author.

## Supplementary material

SUPPLEMENTARY FIGURE S1 Global leadership initiative on malnutrition (GLIM) [reprinted with permission (20)].
