# Supplementary material for: The global leadership initiative on malnutrition criteria for the diagnosis of malnutrition in patients with chronic liver diseases: a systematic review and meta-analysis
Source: Front Nutr. 2025 Jun 19;12:1612417. doi: 10.3389/fnut.2025.1612417 (PMC12222187; doi:10.3389/fnut.2025.1612417)
Supplement: SUPPLEMENTARY FIGURE S1 — Global leadership initiative on malnutrition (GLIM) [reprinted with permission (20)]. [file Table_1.docx]

**TABLE S1**

Literature Search Strategy for Meta-Analysis Based on GLIM and CLD.

| **Search Concept** | **Keywords (MeSH Terms + Free-text Terms)** | **Boolean Operators Combination** |
| --- | --- | --- |
| GLIM-related | ("Global Leadership Initiative on Malnutrition"[MeSH] OR "GLIM criteria"[tiab] OR "malnutrition diagnosis"[tiab] OR "GLIM framework"[tiab]) | (GLIM[tiab] OR "nutritional assessment"[tiab] OR "diagnostic criteria"[tiab]) Combined with OR |
| CLD-related | ("Chronic Liver Disease"[MeSH] OR "CLD"[tiab] OR "hepatic disease"[tiab] OR "liver cirrhosis"[tiab]) | (CLD[tiab] OR "liver fibrosis"[tiab] OR "hepatic insufficiency"[tiab]) Combined with OR |
| Combined Search Strategy | (GLIM-related search terms) AND (CLD-related search terms) | Final combination using AND to connect all concepts |

GLIM, Global Leadership Initiative on Malnutrition; CLD, chronic liver disease.
