# Supplementary figures and images for: The global leadership initiative on malnutrition criteria for the diagnosis of malnutrition in patients with chronic liver diseases: a systematic review and meta-analysis
Source: Front Nutr. 2025 Jun 19;12:1612417. doi: 10.3389/fnut.2025.1612417 (PMC12222187; doi:10.3389/fnut.2025.1612417)

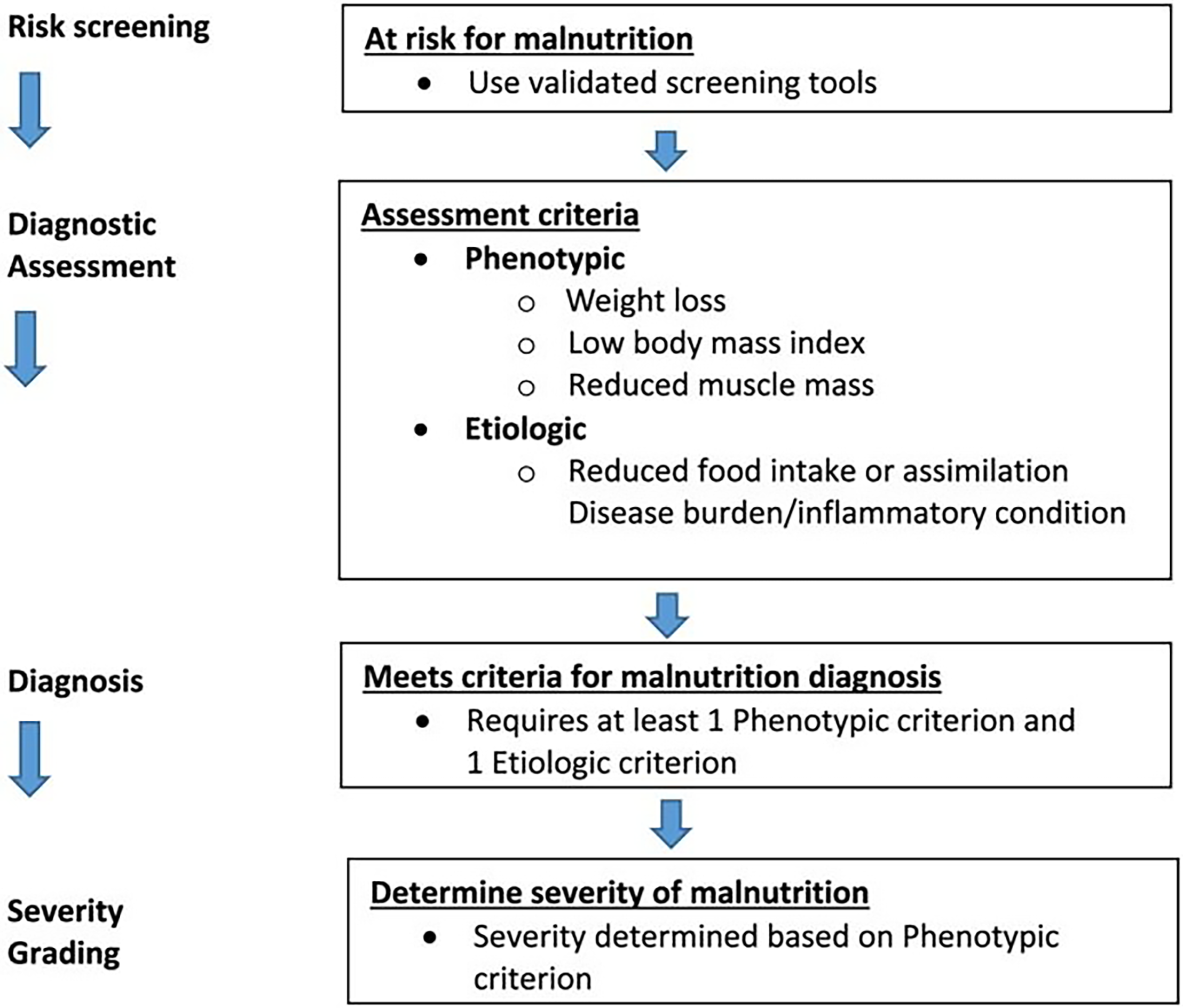

Supplement: Supplementary file 2 [file Image_1.tif]
